# Personalizing Breast Cancer Irradiation Using Biology: From Bench to the Accelerator

**DOI:** 10.3389/fonc.2018.00083

**Published:** 2018-04-05

**Authors:** David Azria, Muriel Brengues, Sophie Gourgou, Celine Bourgier

**Affiliations:** ^1^Institut de Recherche en Cancérologie de Montpellier (IRCM), INSERM U1194, Montpellier, France; ^2^Université de Montpellier, Montpellier, France; ^3^Institut Régional du Cancer de Montpellier (ICM), Montpellier, France

**Keywords:** radiosensitization, normal tissue complications, predictive factors, biomarkers, breast cancer

## Abstract

While adjuvant treatments of early breast cancers (BCs) had significantly improved patients’ overall survival, some of them will still develop locoregional relapses and/or severe late radio-induced toxicities. Here, we propose to review how to personalize locoregional treatment by identifying patients at high and low risk of locoregional relapse, patients at risk of late radio-induced side effects. We will, therefore, discuss how to enhance BC radiosensitivity. Finally, we will address how personalized radiotherapy could be implemented in prospective clinical trials.

## Introduction

In France, incidence of early breast cancer (BC) is 54,062 in 2015 (data from FRANCIM, French network of cancer registries). Adjuvant treatments of early stage BC had significantly improved patients’ overall survival (OS) over time. In patients younger than 75 years, the French 5-years OS is 91–93% in 2005–2010 period versus 83–88% in 1994–1998 period. Similar observations were noticed worldwide, and this improvement could be attributable to mammography and advances in treatment. Indeed, in the last two decades, biological tumor analysis permitted to classify BC prognostic outcomes according to molecular classification: luminal (positive estrogen and/or progesterone receptors—ER/PgR), triple negative (negative ER/PgR/negative Her2), and Her2 overexpression phenotypes (positive Her2) (1, 2). Since the introduction of BC molecular classification, systemic therapies are tailored according to BC heterogeneity (biomarkers as ER/PgR, Her2, Ki67) and aggressiveness (histological subtypes, histological grade, and tumor stage). Different methods have been developed to help clinicians in BC management as prognostic tools (Adjuvant! Online, Predict, the Nottingham Prognostic Index); as commercialized mRNA based gene classifiers [OncotypeDx^®^/Genomic Health; MammaPrint^®^/Agendia; MapQuantDxTM (Genomic Grade Index—GGI)/Ipsogen/QIAGEN; ProSigna^®^/nanoString; EndoPredict^®^/Sividon/Myriad Genetics].

Nevertheless, personalizing treatments in the field of locoregional treatment is less developed. Here, we propose to review which BC population would be at high risk of locoregional relapse, the trends of radiosensitization in BC, and how to identify patients at risk of late radio-induced side effects.

## Predictive Biomarkers for BC Radiotherapy

### Current Prognostic Factors of Locoregional Recurrences

Adjuvant BC radiotherapy reduced both recurrence and BC mortality after mastectomy and axillary lymph nodes dissection (3) and after breast conserving surgery (BCS) (4). Usual prognostic factors of locoregional relapses (LRR) reported in the literature were young age, axillary lymph nodes involvement, tumor size, involved margins, histological grade, negative estrogen receptor, and presence of extensive ductal *in situ* and lymphovascular invasion (5). A recent meta-analysis showed that patients with luminal-A subtype BC displayed lesser LRR than triple-negative (TN) or Her2-positive BC (6). Those results are consistent with others reported in the literature such as Braunstein and colleagues’ study (7): among 2,233 BC patients treated from 1998 to 2007, a total of 69 local relapse were observed with a median follow-up of 106 months. They observed that non luminal-A BCs were significantly at higher risk of local relapses with a hazard ratio (HR) at 2.64 for luminal-B, at 5.42 for Her2-enriched, and at 4.32 for triple negative BCs, respectively (*p* < 0.001 for each).

### Luminal-A versus Non Luminal-A BCs: Is Molecular Subtype Predictive of Radiotherapy Efficacy?

#### Luminal-A BC and Patients at Low Risk of Local Relapse

Patients’ population enrolled in the Toronto–British Columbia trial was luminal-A BC phenotype and was considered at low risk of local relapse (older than 50 years, T1-2 and node-negative BC) (Table 1). They were randomly assigned to tamoxifen or tamoxifen and breast RT (8). A recent molecular phenotypes analysis through tissue micro-arrays (TMA) was performed in order to determine whether an intrinsic subtyping would be a predictive biomarker of adjuvant BC radiotherapy benefit (9). Luminal A subtype was defined as followed: positive ER and/or PgR (immunostaining seen in >1% of tumor nuclei), negative Her2 and Ki67 < 14% (10, 11). A total of 501 formalin-fixed paraffin-embedded blocks were retrieved among the 769 patients enrolled. The median follow-up was 10 years, and the 10y-OS was 84% in both arms. Usual clinical and pathological factors were studied (age, tumor size, tumor grade) as well as BC phenotypes (luminal-A, luminal-B, basal-like, and Her2-enriched BC). Luminal-A BC patients significantly displayed a lower risk of local relapse compared to other phenotypes with a 10y-ipsilateral breast tumor recurrence (IBTR) at 5.2%. While luminal-A and -B BCs seemed to have a lesser benefit from adjuvant BC radiotherapy (luminal-A, HR = 0.40; 95% confidence interval = 0.12–1.29; and luminal-B, HR = 0.51; 95% CI = 0.19–1.36), no significant interaction was observed between molecular phenotype and treatments.

**Table 1:** Risk of ipsilateral breast tumor recurrence (IBTR) in luminal A BC patients.

Trials	Patients population		IBTR in luminal breast cancer (BC)	Reduction risk [hazard ratio (HR)]	
Toronto British Columbia	All patients		10 years-IBTR = 5.2%	HR = 0.40 (95% CI = 0.12–1.29)	No statistical interaction radiotherapy effect and molecular subtypes

SweBCG91 (breast conserving surgery)	All Lum-A patients	W/o adjuvant RT	10 years-IBTR = 19%	HR = 0.46 (95% CI = 0.29–0.74)	
		With adjuvant RT	10 years-IBTR = 9%	*p* = 0.001	

	Lum-A, >65 years, pN0	W/o adjuvant RT	10 years-IBTR = 20%	HR = 0.30 (95% CI = 0.11–0.81)	
		With adjuvant RT	10 years-IBTR = 6%	*p* = 0.008	

BC and Danish Breast Cancer Group (DBCG)-82b	BC	W/o adjuvant RT	20 years-IBTR = 31%	0.17 (95% CI = 0.01−0.92) *p* = 0.04	

	DBCG-82b	W/o adjuvant RT	20 years-IBTR = 42%	0.12 (95% CI = 0.01−1.02) *p* = 0.05	

Molecular phenotype in such patients population is a significant prognostic factor of IBTR, but not predictive. The recent report of a meta-analysis showed similar findings (6).

#### Patients Treated With BCS and LUMINAL-A BC

The Swedish Breast Cancer Group 91 Radiotherapy Randomized Clinical Trial (SweBCG91-RT trial) assigned patients to undergo BCS alone versus BCS followed by adjuvant radiotherapy in node-negative stage I-II BC patients (12). Systemic treatments, chemo or hormone therapy, were mostly omitted (92% of patients). A total of 958 TMA was performed among the 1,003 patients enrolled (95% of patients population) and tumor subtypes were defined according to the St. Gallen International Breast Cancer Conference (2013) Expert Panel (13). BC subtypes were prognostic for time to IBTR with a shorter interval time of relapse for TN and Her2-enriched BC (mostly in the first 5 years of follow-up).

After a median follow-up of 15 years, adjuvant radiotherapy significantly decreased the risk of 10y-IBTR as first event in luminal-A (from 19 to 9%; HR = 0.46; 95% CI = 0.29–0.74; *p* = 0.001) and luminal-B (non Her2 enriched; from 24 to 8%; HR = 0.33; 95% CI = 0.16–0.65; *p* = 0.001) phenotypes but not for Her2-enriched (from 15 to 19%; HR = 1.29; 95% CI = 0.38–4.40; *p* = 0.6), and triple negative BC patients (from 21 to 6%; HR = 0.25; 95% CI = 0.05–1.12; *p* = 0.08). A subpopulation of patients were defined at low risk (age over than 65 years, N0 and luminal A molecular subtype). Among those patients, the risk of IBTR was not as low as expected with a cumulative incidence of IBRT as first event at 10 years at 20% in absence of radiotherapy and at 6% in presence of radiotherapy (HR = 0.30; 95% CI = 0.11–0.81; *p* = 0.008). However, BC subtype was not predictive of response to radiotherapy (*p* = 0.21 for interaction tests).

#### Patients Treated With Radical Mastectomy and Luminal-A BC

A recent study included The British Columbia randomized radiation trial and the Danish Breast Cancer Group (DBCG) protocol 82b assessed whether intrinsic subtypes would be predictive on postmastectomy radiotherapy (PMRT) efficacy regarding LRR in young premenopausal and lymph node-positive BC patients (14). After 20 years of follow-up, the cumulative incidence of LRR in the entire cohort and in absence of PMRT was 36 and 42% in British Columbia and DBCG-82b trials, respectively. During time, PMRT significantly decreased LRR risk in both clinical trials with a HR at 0.35 (95% CI = 0.17−0.72; *p* = 0.004) in the British Columbia and HR at 0.30 (95% CI = 0.11−0.83; *p* = 0.02) in the DBCG-82b trials, respectively. As luminal-A BCs are considered at low risk of LRR, the authors studied their cumulative incidence in absence of PMRT: 31 and 42% of LRR at 20 years in British Columbia and DBCG-82b trials, respectively. Authors tested whether PMRT was useful in luminal-A patients’ cohort and they observed that PMRT significantly decreased LRR risk in such patients with a HR at 0.17 (95% CI = 0.01−0.92; *p* = 0.04) and at 0.12 (95% CI = 0.01−1.02; *p* = 0.05) in British Columbia and DBCG-82b trials, respectively. The low numbers of patients in each subgroup is one of the major limitations of this study, and therefore, no robust conclusions could be stated.

In conclusion, to date, BC subtypes are not predictive of adjuvant BC radiotherapy regardless the type of surgery, while molecular subgroups are associated with LRR risk. Therefore, in daily practice, no predictive tool could be used to personalized adjuvant radiotherapy in BC settings.

### Perspectives in Personalization of Adjuvant BC Radiotherapy

Torres-Rocca has developed a 10 genes-signature, namely RadioSensitive Index (RSI), which estimates BC radiosensitivity (low RSI = radiosensitive profile/RSI-S; high RSI = radioresistant profile/RSI-R; intermediate RSI/RSI-I) (15). In patients treated by surgery and adjuvant radiotherapy, RSI was related to patients’ outcome: radiosensitive patients displayed a significant higher relapse-free survival and distant metastases-free survival at 5 years (5 years RFS = 95%; 5 years DMFS = 77%) than radioresistant patients (5 years RFS = 75%; 5 years DMFS = 64%) [5 years RFS: HR = 7.47 (95% CI = 1–56.01), *p* = 0.02; 5 years DMFS: HR = 1.74 (95% CI = 1.02–2.99), *p* = 0.049]. In absence of adjuvant RT, the RSI signature was not able to distinguish patients at risk to develop relapses regardless the type of surgery (lumpectomy or mastectomy) (15). When RSI was combined to molecular subtypes defined according to Eroles and colleagues (16), the authors observed that local relapse was significantly increased in triple negative and RSI-R BC with a HR at 0.37 (95% CI = 0.15–0.92; *p* = 0.02) (17). Interestingly, when triple negative BC had a sensitive or intermediate RSI, patients’ outcome reached luminal BC outcome. Even though no impact of RSI was observed among the entire cohort of luminal BCs in terms of patients’ outcome, a dose effect was noticed in those latter patients population. Hence, increasing total dose by a boost of 16 Gy to tumor bed significantly improved patients’ outcome with RSI-R luminal BC with a 5 years-local relapse survival at 91 versus 78% (no boost).

As triple negative BCs displayed a higher risk of LRR, some preclinical studies assessed whether the addition of a radiosensitizer would increase response to ionizing radiation in those BCs. For instance, Yes-associated protein 1 (YAP1), which is the terminal effector of the Hippo pathway is involved in TNBC radioresistance. The use of verteporfin, a YAP1 inhibitor, radiosensitized TNBC cell lines by inhibiting EGFR/PI3K/AKT signaling pathway and by increasing DNA damages (18). Other preclinical approaches have been developed as the use of niclosamide, a potent inhibitor of Wnt/β-catenin signaling (19). Yin and colleagues observed that niclosamide radiosensitized TNBC cell lines *in vitro* by increasing radio-induced apoptosis and induced tumor growth delay *in vivo*.

In addition to TNBC, Her2-enriched BC also displayed a poor prognosis and a high LRR risk. More than one decade ago, Pietras and colleagues showed that Her2 inhibition radiosensitized BC cell lines by increasing radio-induced DNA damages and apoptosis (20). When BC cells displayed positive Her2 phenotype associated to BC stem cells phenotype (i.e., CD44^+^CD24^low^), those cells showed an aggressiveness and radioresistant phenotype (21). When those cells were in presence to trastuzumab, a specific Her2 antibody, their aggressiveness, and radioresistance significantly decreased. More recently, Hou and colleagues showed that Her2 was involved in BC radioresistance by promoting focal adhesion kinase (Fak) phosphorylation and thereby by promoting epithelial-to-mesenchymal transition (22). Therefore, targeting Fak would be a potential target for Her2 BC radiosensitization.

## Radio-Induced Toxicity Biomarkers ERA

Toxicities after adjuvant BC radiotherapy, such as a poor cosmetic outcome, can have a negative impact on quality of life and a marked effect on subsequent psychological outcome. Severe toxicities will still occur in 5–10% of patients treated by 3D-conformal radiotherapy, such as radio-induced fibrosis (RIF), even though in the respect of dose–volume constraints to organs-at-risk (23, 24). Furthermore, owing to considerable progress in cancer management in recent decades, the number of long-term survivors significantly increased in BC patients’ population. In that context, many normal tissue radiosensitivity assays were developed to predict the risk of late toxicities occurrence.

### Fibroblast-Based Assays

Clinical onset of severe radio-induced toxicities in children with ataxia telangiectasia (AT) was the starting point to develop assays to predict intrinsic radiosensitivity (19, 25). AT is an autosomal recessive disorder related to *ATM* gene mutations, which is involved in DNA damage repair. Several fibroblast-based assays were developed to predict late radio-induced toxicities occurrence as well as clonogenic assays, residual double strand breaks, micronuclei formation, and comet assays (19, 23–25). The rational was that irradiation-induced DNA damage leads to cell death and, therefore, was the mirror of normal tissue response to ionizing radiation exposure. Even though those tests showed promising results, none of them has been validated in a large clinical trial (26–28). More recently, a new ATM protein-based assay has been developed from patients’ skin biopsy in patients who had developed severe radio-induced toxicities (29). Those latter patients displayed a delay in nucleoshuttling of the ATM protein in response to ionizing radiation of normal tissue. To date, no prospective study showed that one of those assays was able to predict severe late toxicities occurrence (26, 27).

### Lymphocytes-Based Assays

Since 1995, a rapid (72 h) radiosensitivity assay was developed and based on flow cytometric assessment of radiation-induced CD8 T-lymphocyte apoptosis (RILA) (28). RILA was first compared between AT and healthy children. A low RILA value was significantly associated with AT syndrome (29, 30). Based on this exploratory study, RILA value was retrospectively assessed in patients with DNA repair-related diseases (i.e., Nijmegen breakage and AT syndromes). Those patients with individual radiosensitivity also displayed a low RILA rate. Therefore, a prospective study included miscellaneous cancers was conducted to determine whether RILA assay would be helpful to identify patients at high risk of severe radio-induced toxicities. This study concluded that patients with severe toxicities displayed a compromised apoptotic response (31) as well as other studies which drew similar conclusions (32–35). The predictive value of RILA in RIF occurrence was validated within the PHRC2005 (NCT00893035), a prospective multicenter French study published in December 2015 (36). In this study, a significant relationship between RILA and toxicities occurrence was observed: an increased RIF development was significantly related to RILA.

Based on the data from the PHRC2005 (NCT00893035), a multiparametric nomogram was developed. The nomogram incorporates both the RILA value, as a continuous variable, and other independent parameters related to the patient’s environment and treatment. This virtual biomarker has shown to significantly improve the performance of the RILA assay alone (*p* < 0.0013). The prediction of RIF, based on the RILA assay, should now include the nomogram analysis.

In the meanwhile, other assays were developed since many years such as residual γH2AX foci, G2 metaphase, G0 micronuclei assays or GWAS, SNPs studies. Most of these assays-related studies are retrospective or in case of prospective studies, results were non-statistically significant [reviewed in Ref (37, 38)]. For instance, the last published study compared four assays in a recent small cohort (*n* = 12). Even though this study was very small, RILA still performed best (39).

### Validity of Predictive Assays According to REMARK Guidelines

According to REMARK guidelines (40, 41), RILA get the highest level of evidence (level I) while level of evidence is quite low for others assays (level III or IV).

## Clinical Implementation

As stated by Azria and colleagues (42), personalized radiotherapy could be address in prospective clinical trials according to tumor control (LRR^high^ or LRR^low^) and normal tissue complications (RIF^high^ or RIF^low^) probability (Table 2).

**Table 2:** Proposed prospective clinical trials according to tumor control (LRR^high^ or LRR^low^) and normal tissue complications (RIF^high^ or RIF^low^) probability.

Risk of locoregional relapse (LRR) and severe fibrosis (RIF)	Proposed personalized radiotherapy
LRR^low^/RIF^low^	Hypofractionated radiotherapy
LRR^low^/RIF^high^	Partial breast irradiation or radiotherapy omission
LRR^high^/RIF^low^	Dose escalation scheme
LRR^high^/RIF^high^	Tumor control should be mandatory combined to mitigation treatment

For instance, BC patients at LRR^low^/RIF^low^ risk, hypofractionated radiotherapy schedule could be proposed. In case of patients at LRR^low^/RIF^high^ risk, the authors suggested either a more conformal radiotherapy, as accelerated partial breast irradiation, or an alternative approach by using surgery to avoid radiotherapy.

For patients at LRR^high^/RIF^low^ risk, dose escalation, such as a greater boost dose or irradiation field extensions could be considered to improve local control without severe toxicities, providing enhanced clinical benefit. For patients at LRR^high^/RIF^high^ risk, tumor control should be mandatory. In those latter patients, mitigation treatment could be proposed when late and severe toxicities will occur such as the use of pentoxifyllin–tocopherol combination (43, 44) or the use of statins (45).

## Author Contributions

All authors participated in conception of the review, drafting and revising the article, and providing approval for publication of the content.

## Conflict of Interest Statement

The authors declare that the research was conducted in the absence of any commercial or financial relationships that could be construed as a potential conflict of interest.
